# Modeling Inhibitory Effect on the Growth of Uninfected T Cells Caused by Infected T Cells: Stability and Hopf Bifurcation

**DOI:** 10.1155/2018/3176893

**Published:** 2018-08-12

**Authors:** Yahui Ji, Wanbiao Ma, Keying Song

**Affiliations:** Department of Applied Mathematics, School of Mathematics and Physics, University of Science and Technology Beijing, 100083, Beijing, China

## Abstract

We consider a class of viral infection dynamic models with inhibitory effect on the growth of uninfected T cells caused by infected T cells and logistic target cell growth. The basic reproduction number *R*_0_ is derived. It is shown that the uninfected equilibrium is globally asymptotically stable if *R*_0_ < 1. Sufficient conditions for the existence of Hopf bifurcation at the infected equilibrium are investigated by analyzing the distribution of eigenvalues. Furthermore, the properties of Hopf bifurcation are determined by the normal form theory and the center manifold. Numerical simulations are carried out to support the theoretical analysis.

## 1. Introduction

The human immunodeficiency virus (HIV) is a lentivirus, which replicates by infecting and destroying primarily CD4^+^ T cells. The end stage of HIV viral progression is acquired immune deficiency syndrome (AIDS) (see, for example, [1]), identified when the count of individual's CD4^+^ cells count falls below 200. Since AIDS was found in America in 1981, it spread worldwide and became the public health and social problem which causes serious damage to human survival and development. In 2016, there exist about 38 million people living with human immunodeficiency virus (HIV) (see, for example, [2]). Thus, it is a challenge to study and control the virus.

It is widely known that mathematical models have made considerable contributions to understanding the HIV infection dynamics. Nowak et al. have proposed a class of classic mathematical model to describe HIV infection dynamics (see, for example, [3–6]),

(1) x˙t=s−dxt−βxtvt,y˙t=βxtvt−pyt,v˙t=kyt−uvt,

where *x*(*t*), *y*(*t*), and *v*(*t*) denote the concentrations of uninfected cells, infected cells, and free virus at time *t*, respectively. Uninfected cells are produced at the rate *s*  (*s* > 0), die at the rate *d*  (*d* > 0), and become infected at the rate *β*  (*β* > 0). The constant *p*  (*p* > 0) is the death rate of the infected cells due either to virus or to the immune system. The constant *k*  (*k* > 0) is the rate of production of virus by infected cells and the constant *u*  (*u* > 0) is the rate at which the virus is cleared.

Incorporating the life cycle of the virus in the cells, some researchers have considered that the HIV virus from HIV infection to produce new virus takes time. To make a better understanding for this phenomenon in mathematics, HIV models including time delay have been proposed (see, for example, [4, 7–9]). Several researchers have considered that when T cells stimulate by antigen or mitogen, this will differentiate and increase in the number. The HIV model with a full logistic mitosis term has been investigated (see, for example, [6, 10, 11]). Taking into account the growth of uninfected cells, they made a further investigation to add a full logistic term *rx*(*t*)(1 − (*x*(*t*) + *y*(*t*))/*T*) (see, for example, [12, 13]).

In the above model, there are two factors that accelerate the reduction of uninfected cells: one is the natural death of uninfected cells and the other is that uninfected cells become infected cells. HIV gene expression products can be toxic and directly or indirectly induce apoptosis in uninfected cells. Some data show that viral proteins interact with uninfected cells and produce an apoptotic signals that accelerate the death of uninfected cells. Recently, Wang and Zhang proposed a spatial mathematical model to describe the predominance for driving CD4^+^ T cells death, which is called caspase-1-mediated pyroptosis (see, for example, [14]).

Based on model (1), Guo and Ma have proposed a class of delay differential equations model of HIV infection dynamics with nonlinear transmissions and apoptosis induced by infected cells (see, for example, [15]). And then, Cheng et al. [16] have considered the following infection model with inhibitory effect on the growth of uninfected cells by infected cells:

(2) x˙t=s−dxt−cxtyt−βxtvt,y˙t=δxt−τvt−τ−pyt,v˙t=kyt−uvt,

where the constant *c*  (*c* > 0) represents the rate of apoptosis at which infected cells induce uninfected cells. *δ*  (*δ* > 0) denotes the surviving rate of infected cells before they become productively infected. The biological meanings of the other parameters in the model (2) are similar to that in the model (1).

Motivated by the above models, in this paper, we will study a delay differential equation model of HIV infection with a full logistic term of uninfected cells,

(3) x˙t=s+rx1−xt+ytT−dxt−cxtyt−βxtvt,y˙t=δxt−τvt−τ−pyt,v˙t=kyt−uvt.

In this model, the logistic growth of the healthy CD4^+^ T cells is described by *rx*(*t*)(1 − (*x*(*t*) + *y*(*t*))/*T*). The total concentration of CD4^+^ T cells is *x*(*t*) + *y*(*t*), where *x*(*t*) denotes the concentration of uninfected cells, *y*(*t*) is the concentration of infected cells, and *T* is the maximum level of CD4^+^ T cells. *δ*  (*δ* > 0) is the infection rate of infected cells. The biological meanings of the other parameters in the model (3) are similar to that in the model (2).

The main purpose of this paper is to carry out a pretty theoretical analysis on the stability of the equilibria of the model (3) and to analyze the Hopf bifurcation by related theories of the differential equations. The organization of this paper is as follows. In Section 2, we investigate the existence and the ultimate boundedness of the solutions of the model (3). Then we consider the global stability of the uninfected equilibrium and the Hopf bifurcation at the infected equilibrium. In Section 3, some properties of Hopf bifurcation such as direction, stability, and period are determined. In Section 4, the brief conclusions are given and sets of numerical simulations are provided to illustrate the main results.

## 2. Local and Global Stability of the Equilibria

According to biological meanings, we assume that the initial condition of the model (3) is given as follows:

(4) xθ=ϕ1θ,yθ=ϕ2θ,vθ=ϕ3θθ∈−τ,0,

where *ϕ* = (*ϕ*_1_, *ϕ*_2_, *ϕ*_3_)^*T*^ ∈ *C* such that *ϕ*_*i*_(*θ*) ≥ 0  (*i* = 1,2, 3). Here, *C* = *C*([−*τ*, 0]; *R*_+_^3^) denotes the Banach space of continuous functions mapping from the interval [−*τ*, 0] to *R*_+_^3^ equipped with the supnorm.

The existence and uniqueness, nonnegativity, and boundedness of the solutions of the model (3) with the initial condition (4) can be given as follows.

**Theorem 1 .**

The solution (*x*(*t*), *y*(*t*), *v*(*t*)) of the model (3) with the initial condition (4) is existent, unique, and nonnegative on [0, +*∞* and also has

(5) lim sup t→+∞xt≤x0,lim sup t→+∞xt+yt+τ≤s+rx0d~,lim supt→+∞ vt≤ks+rx0ud~,

where $\tilde{d}=min\left\{d,p\right\}$ and $x_{0}=\left(T/2r\right)\left(r-d+\sqrt{{\left(r-d\right)}^{2}+4sr/T}\right)$.

In fact, by using standard theorems for existence and uniqueness of functional differential equations (see, for example, [17–19]), we can show that the solution (*x*(*t*), *y*(*t*), *v*(*t*)) of the model (3) with the initial condition (4) is existent, unique and nonnegative on [0, +*∞*, easily. And the proving of ultimately bounded of the solution (*x*(*t*), *y*(*t*), *v*(*t*)) is similar to [12, 16].

We can denote the basic reproduction number of the HIV virus for the model (3) as *R*_0_ = (*kδ*/*pu*)*x*_0_ (see, for example, [3]). For the existence of nonnegative equilibria of the model (3), we can obtain the following classifications:

(i) The model (3) always has the uninfected equilibrium *E*_0_ = (*x*_0_, 0,0).

(ii) If *R*_0_ = (*kδ*/*pu*)*x*_0_ > 1, the model (3) has unique infected equilibrium *E*^*∗*^ = (*x*^*∗*^, *y*^*∗*^, *v*^*∗*^), where

(6) x∗=puδk,y∗=ukv∗,v∗=−rx∗2/T+r−dx∗+srx∗u/kT+cu/kx∗+βx∗.

**Theorem 2 .**

If *R*_0_ < 1, the uninfected equilibrium *E*_0_ of the model (3) is globally asymptotically stable.

**Proof**

We consider linear system of the model (3) at *E*_0_, we have

(7) x˙t=r−d−2rTx0xt−rT+cx0yt−βx0vt,y˙t=δx0vt−τ−pyt,v˙t=kyt−uvt.

The corresponding characteristic equation is given by

(8) $$\begin{array}{c} \left(\;\lambda -r+d+\frac{2r}{T}x_{0}\;\right)\left[\;\left(\;\lambda +p\;\right)\left(\;\lambda +u\;\right)-k\delta x_{0}e^{-\lambda \tau }\;\right]=0. \end{array}$$

Clearly, one of the roots is $\lambda_{1}=r-d-\left(2r/T\right)x_{0}=-\sqrt{{\left(r-d\right)}^{2}+4rs/T}<0$, so the local stability depends on the other two roots generated by

(9) $$\begin{array}{c} \lambda^{2}+\left(\;p+u\;\right)\lambda +pu-k\delta x_{0}e^{-\lambda \tau }=0. \end{array}$$

When *R*_0_ < 1, *pu* − *kδx*_0_ ≠ 0. Therefore, *λ* = 0 is not root of (9). If (9) has pure imaginary root *λ* = *iω*  (*ω* > 0) for some *τ* > 0, substituting it into (9) and separating the real and imaginary parts, it has

(10) pu−w2=kδx0cos⁡wτ,p+uw=−kδx0sin⁡wτ.

It follows that

(11) $$\begin{array}{c} f\left(\;\tilde{\omega }\;\right)\equiv {\tilde{\omega }}^{2}+\left(\;p^{2}+u^{2}\;\right)\tilde{\omega }+p^{2}u^{2}-k^{2}\delta^{2}x_{0}^{2}=0, \end{array}$$

where $\tilde{\omega }=\omega^{2}$. Since *p*^2^ + *u*^2^ > 0, *p*^2^*u*^2^ − *k*^2^*δ*^2^*x*_0_^2^ = *p*^2^*u*^2^(1 − *R*_0_^2^) > 0, we have $f(\tilde{\omega })>0$, which contradicts $f(\tilde{\omega })=0$. This suggests that all the roots of (8) have negative real parts for any time delay *τ* ≥ 0. Therefore, the uninfected equilibrium *E*_0_ of the model (3) is locally asymptotically stable.

Define

(12) G=ϕ=ϕ1,ϕ2,ϕ3∈C ∣ 0≤ϕ1≤x0,  ϕ2≥0,  ϕ3≥0.

It is easy to show that *G* attracts all solutions of the model (3) and is also positively invariant with respect to the model (3).

Motivated by the methods in [20, 21], we choose the following Liapunov functional:

(13) $$\begin{array}{c} L\left(\;\phi \;\right)=\frac{1}{\delta }\phi_{2}\left(\;0\;\right)+\frac{p}{\delta k}\phi_{3}\left(\;0\;\right)+\overset{0}{\underset{-\tau }{\int }}\phi_{1}\left(\;\theta \;\right)\phi_{3}\left(\;\theta \;\right)d\theta \end{array}$$

for any *ϕ* ∈ *G*. The time derivative of *L* along the solutions of the model (3) is

(14) L˙1δy′t+pδkv′t+xtvt−xt−τvt−τ=xt−upkδvt≤x0−upkδvt=1−1R0x0vt≤0,

where *t* ≥ 0. By using Liapunov-LaSalle invariance principle [18], the uninfected equilibrium *E*_0_ of the model (3) is globally asymptotically stable.

Next, let us study the stability of the infected equilibrium *E*^*∗*^. The linearized system of the model (3) at *E*^*∗*^ is

(15) ddtxt=−sx∗+rx∗Txt−rx∗Tyt−βx∗vt−cx∗yt,ddtyt=δx∗vt−τ+xt−τv∗−pyt,ddtvt=kyt−uvt.

Denote

(16) B=sx∗+rx∗T,E=rT+cx∗,F=βx∗,G=δv∗,H=δx∗.

The corresponding characteristic equation is

(17) λ3+B+p+uλ2+Bp+uB+upλ+uBp+EG−kHλ+kGF+uEG−kBHe−λτ=0.

Define

(18) a1=B+p+u>0,a2=Bp+uB+up>0,a3=uBp>0,b2=EG−kH,b3=kGF+uEG−kBH,

where *b*_2_ = *pu*(*rv*^*∗*^/*kT* − 1) + *cδx*^*∗*^*v*^*∗*^ and *b*_3_ = *pu*(*βv*^*∗*^ + *ruv*^*∗*^/*kT* − *B*) + *cδux*^*∗*^*v*^*∗*^.

Therefore, (17) becomes

(19) $$\begin{array}{c} \lambda^{3}+a_{1}\lambda^{2}+a_{2}\lambda +a_{3}+\left[\;b_{2}\lambda +b_{3}\;\right]e^{-\lambda \tau }=0. \end{array}$$

When *τ* = 0, (19) becomes *λ*^3^ + *a*_1_*λ*^2^ + (*a*_2_ + *b*_2_)*λ* + (*a*_3_ + *b*_3_) = 0. Notice that *a*_1_ > 0, *a*_3_ + *b*_3_ = *pu*(*βv*^*∗*^ + *ruv*^*∗*^/*kT*) + *cδux*^*∗*^*v*^*∗*^ > 0. Thus, if *R*_0_ > 1 and Δ_2_ = *a*_1_(*a*_2_ + *b*_2_)−(*a*_3_ + *b*_3_) > 0 hold, by Routh-Hurwitz criterion, the infected equilibrium *E*^*∗*^ is locally asymptotically stable when *τ* = 0.

Now, let us investigate the stability of *E*^*∗*^ when *τ* > 0. Rewriting (19) as

(20) $$\begin{array}{c} P\left(\;\lambda \;\right)+Q\left(\;\lambda \;\right)e^{-\lambda \tau }=0, \end{array}$$

where

(21) Pλ=λ3+a1λ2+a2λ+a3,Qλ=b2λ+b3.

Since *a*_3_ + *b*_3_ = *uBp* + *pu*(*βv*^*∗*^ + *ruv*^*∗*^/*kT* − *B*) + *cδux*^*∗*^*v*^*∗*^ > 0, *λ* = 0 is not the root of (19). Assume that (19) has pure imaginary *λ* = *iw*  (*w* > 0) for some *τ* > 0; substituting it into (19), it has −*iw*^3^ − *a*_1_*w*^2^ + *ia*_2_*w* + *a*_3_ + (*ib*_2_*w* + *b*_3_)(cos *wτ* − *i* sin *wτ*) = 0, and separating the real and imaginary parts, we have

(22) w3−a2w=b2wcos⁡wτ−b3sin⁡wτ,a1w2−a3=b2wsin⁡wτ+b3cos⁡wτ.

Therefore, it has

(23) $$\begin{array}{c} w^{6}+c_{1}w^{4}+c_{2}w^{2}+c_{3}=0, \end{array}$$

where *c*_1_ = *a*_1_^2^ − 2*a*_2_, *c*_2_ = *a*_2_^2^ − 2*a*_1_*a*_3_ − *b*_2_^2^, *c*_3_ = *a*_3_^2^ − *b*_3_^2^. Denote *v* = *w*^2^; (23) becomes

(24) $$\begin{array}{c} v^{3}+c_{1}v^{2}+c_{2}v+c_{3}=0. \end{array}$$

Define

(25) $$\begin{array}{c} h\left(\;v\;\right)=v^{3}+c_{1}v^{2}+c_{2}v+c_{3}, \end{array}$$

hence *h*′(*v*) = 3*v*^2^ + 2*c*_1_*v* + *c*_2_. Considering

(26) $$\begin{array}{c} 3v^{2}+2c_{1}v+c_{2}=0. \end{array}$$

It has two real roots, given as $v_{1}=\left(-c_{1}+\sqrt{\Delta }\right)/3$ and $v_{2}=\left(-c_{1}-\sqrt{\Delta }\right)/3$, where Δ = *c*_1_^2^ − 3*c*_2_.

Now, we will illustrate the following conclusions, and it has been proved in [22].

**Lemma 3 .**

For the polynomial (24), the following conclusions are given:

- If *c*_3_ < 0, (24) has at least one positive root.
- If *c*_3_ ≥ 0 and Δ < 0, (24) has no real root.
- If *c*_3_ ≥ 0 and Δ > 0, if and only if $v_{1}=\left(-c_{1}+\sqrt{\Delta }\right)/3>0$ and *h*(*v*_1_) ≤ 0, (24) has real roots.

Assume that *h*(*v*) = 0 has positive real roots. Generally, we may suppose that (24) has *k*  (1 ≤ *k* ≤ 3) positive real roots, denoted as *v*_1_, *v*_2_, and *v*_3_. Then, (23) has positive real roots $\omega_{k}=\sqrt{v_{k}}$. From (22), we attain

(27) $$\begin{array}{c} \cos\omega \tau =\frac{b_{2}w^{4}+\left(a_{1}b_{3}-a_{2}b_{2}\right)w^{2}-a_{3}b_{3}}{b_{2}^{2}w^{2}+b_{3}^{2}}. \end{array}$$

Then, we get the corresponding *τ*_*k*_^(*n*)^ > 0 such that (19) has pure imaginary *λ* = *iw*_*k*_, where

(28) τkn=1wkarccos⁡b2wk4+a1b3−a2b2wk2−a3b3b22wk2+b32+2nπ,k=1,2,3,  n=0,1,2,….

Define

(29) $$\begin{array}{c} \tau^{*}=\underset{k\in \left[1,2,3\right]}{\min}\left\{\;\tau_{k}^{\left(0\right)}\;\right\}. \end{array}$$

Differentiating the two sides of (19) with respect to *τ*, it follows that

(30) 3λ2+2a1λ+a2dλdτ+b2e−λτdλdτ−τb2λ+b3e−λτdλdτ−λb2λ+b3e−λτ=0.

Thus, we get

(31) dλdτλ=iwk−1=a2−3wk2+2a1wkia2wk2−wk4−a3wk−a1wk3i+b2−b2wk2+b3wki.

Then

(32) dResλdτλ=iwk−1=a2−3wk2a2wk2−wk4−2a1wka3wk−a1wk3a2wk2−wk42+a3wk−a1wk32+−b22wk2−b22wk4+b32wk2.

From (22), we obtain *b*_2_^2^*w*^2^ + *b*_3_^2^ = (*w*^3^ − *a*_2_*w*)^2^ + (*a*_1_*w*^2^ − *a*_3_)^2^. Therefore,

(L) dResλdτλ=iwk−13vk3+2c1vk2+c2vkwk2b22wk2+b32=vkh′vkwk2b22wk2+b32.

Since *v*_*k*_ > 0, we get Re⁡(*dλ*(*τ*)/*dτ*)|_*τ*=*τ*_*k*_^(*n*)^_ and *h*′(*v*_*k*_) have the same sign. Combining Lemma 3 with the above L, we have the following conclusions.

**Theorem 4 .**

*τ*_*k*_^(*n*)^ and *τ*^*∗*^ are defined by (28) and (29). If *R*_0_ > 1, the following results hold:

- If *c*_3_ ≥ 0 and Δ ≤ 0, then infected equilibrium *E*^*∗*^(*x*^*∗*^, *y*^*∗*^, *v*^*∗*^) is locally asymptotically stable.
- If *c*_3_ < 0 or *c*_3_ ≥ 0 and Δ > 0, then infected equilibrium *E*^*∗*^(*x*^*∗*^, *y*^*∗*^, *v*^*∗*^) is locally asymptotically stable when *τ* ∈ [0, *τ*^*∗*^ and unstable when *τ* > *τ*^*∗*^.
- If the conditions of (ii) are all satisfied and *h*′(*v*_*k*_) ≠ 0, then model (3) undergoes a Hopf bifurcation at *E*^*∗*^ when *τ* = *τ*_*k*_^(*n*)^  (*n* = 0,1, 2,…).

## 3. Properties of Hopf Bifurcation

In the above section, we have given the sufficient condition where the model (3) undergoes a Hopf bifurcation at *E*^*∗*^. In this section, we will use the normal form method and the center manifold theory provided in [23, 24] to analysis direction, stability, and the period of the bifurcating periodic solution. By setting *τ* = *τ*^*∗*^ + *μ*, then *μ* = 0 is a Hopf bifurcation value of the model (3). Let *μ*_1_ = *x* − *x*^*∗*^, *μ*_2_ = *y* − *y*^*∗*^, *μ*_3_ = *v* − *v*^*∗*^, and

(33) ut=μ1t,μ2t,μ3tT∈R+3,utθ=ut+θθ∈−τ,0.

Then, the model (3) is equivalent to the functional differential equations ${\dot{u}}_{t}=L_{\mu }(u_{t})+f(\mu ,u_{t})$, defined in *C* : = *C*([−*τ*, 0], *R*_+_^3^), where

(34) fμ,φ=−rTφ120−rT+cφ10φ20−βφ10φ30δφ1−τφ3−τ0.

For *φ* = (*φ*_1_, *φ*_2_, *φ*_3_)^*T*^ ∈ *C*, define *L*_*μ*_*φ* = *Aφ*(0) + *Dφ*(−*τ*). Here,

(35) A=−B−E−F00H000,D=000G−p00k−u.

Using the Riesz representation theorem, there is a 3 × 3 bounded variation matrix function *η*(*θ*, *μ*), which exists for *θ* ∈ [−*τ*, 0], such that *L*_*u*_*φ* = ∫_−*τ*_^0^*dη*(*θ*, *μ*)*φ*(*θ*) holds for any *φ* ∈ *C*. We can choose *η*(*θ*, *μ*) = *Aρ*(*θ*) − *Dρ*(*θ* + *τ*), where

(36) $$\begin{array}{c} \rho \left(\;\theta \;\right)=\left\{\begin{array}{cc} 1, & \theta =0,\\ 0, & \theta \neq 0. \end{array}\right. \end{array}$$

For *φ* ∈ *C*([−*τ*, 0], *R*^3^), define

(37) Aμφ=dφθdθ,θ∈−τ,0,∫−τ0dηs,μφs,θ=0,Rφ=0,θ∈−τ,0,fμ,φ,θ=0.

Then, the system is equivalent to the following operator equation:

(38) $$\begin{array}{c} {\dot{u}}_{t}=A\left(\;\mu \;\right)u_{t}+Ru_{t}. \end{array}$$

Let *C*^*∗*^ = *C*([0, *τ*], (*R*^3^)^*∗*^), and adjoint operator *A*^*∗*^ of *A* is defined by

(39) $$\begin{array}{c} A^{*}\psi \left(\;\xi \;\right)=\left\{\begin{array}{cc} -\frac{d\psi \left(\xi \right)}{d\xi }, & \xi \in \left(0,\tau \right],\\ \overset{0}{\underset{-\tau }{\int }}d\eta \left(s,0\right)\psi \left(-s\right), & \xi =0. \end{array}\right. \end{array}$$

Define the bilinear inner product of *φ* ∈ *C* and *ψ* ∈ *C*^*∗*^ as

(40) ψξ,φθ=ψ¯0φ0−∫θ=−τ0∫s=0θψ¯s−θdηθφsds,

where *η*(*θ*) = *η*(*θ*, 0).

Since *A*(0) and *A*^*∗*^(0) are adjoint operator and ±*iω*^*∗*^ is the eigenvalue of *A*(0), therefore ±*iω*^*∗*^ also is the eigenvalue of *A*^*∗*^. Suppose that the eigenvector of *A*(0) with respect to the eigenvalue *iω*^*∗*^ is *q*(*θ*); the eigenvector of *A*^*∗*^ with respect to the eigenvalue −*iω*^*∗*^ is *q*^*∗*^(*ξ*), and they all satisfy 〈*q*^*∗*^(*ξ*), *q*(*θ*)〉 = 1.

We choose *q*(*θ*) = (1, *q*_2_, *q*_3_)^*T*^*e*^*iω*^*∗*^*θ*^, *θ* ∈ [−*τ*, 0], and $q^{*}\left(\xi \right)=\bar{R}\left(1,q_{2}^{*},q_{3}^{*}\right)e^{i\omega^{*}\xi },\;\;\xi \in [0,\tau ].$ Since *A*(0)*q*(*θ*) = *iω*^*∗*^*q*(*θ*), *A*^*∗*^*q*^*∗*^(*ξ*) = −*iω*^*∗*^*q*^*∗*^(*ξ*), we get

(41) q2=−iω∗+uiω∗+BEiω∗+u+kF,q3=−kiω∗+BEiω∗+u+kF,q2∗=−iω∗−BGeiω∗τ∗,q3∗=Ek+iω∗−Biω∗−skGeiω∗τ∗.

From 〈*q*^*∗*^(*ξ*), *q*(*θ*)〉 = 1 and the similar arguments as in [20–22], we attain the following formula:

(42) $$\begin{array}{c} R={\left[\;1+\bar{q_{2}^{*}}q_{2}+\bar{q_{3}^{*}}q_{3}+\bar{q_{2}^{*}}\tau^{*}\left(\;G+q_{3}H\;\right)e^{-i\omega^{*}\tau^{*}}\;\right]}^{-1}. \end{array}$$

Following the algorithms given in [23] (see, also [13, 24–26]), it then follows that

(43) g20=2R−rT1+q2−cq2−βq3+q2∗¯q3δe−2iω∗τ∗,g11=R−rT2+q2+q2¯−cq2+q2¯−βq3+q3¯+q2∗¯δq3+q3¯,g02=2R−rT1+q2¯−cq2¯−βq3¯+q2∗¯q3¯δe2iω∗τ∗,g21=2R−rT2ω1110+ω2010+ω1120+ω20202+q2−ω20102+q2ω1110−cω1120+ω20202+q2−ω20102+q2ω1110−βω1130+ω20302+q3−ω20102+q3ω1110+q2∗¯δω203−τ∗2eiω∗τ∗+ω203−τ∗eiω∗τ∗+q3¯ω111−τ∗eiω∗τ∗,

where

(44) ω20θ=ig20ω∗q0eiω∗θ+ig02¯3ω∗q¯0e−iω∗θ+E1e2iω∗θ,ω11θ=ig11ω∗q0eiω∗θ+ig11¯ω∗q¯0e−iω∗θ+E2,E1=22iω∗+BEF−Ge−2iω∗τ∗−2iω∗+p−He−2iω∗τ∗0−k2iω∗+u−1×−rT−−rTq2−cq2−βq3δq3e−2iω∗τ∗0,E2=BEF−Gp−H0−ku−1×−2rT−rTq2+q2¯−cq2+q2¯−βq3+q3¯δq3+q3¯0.

Then we can obtain the following quantities:

(45) C10=i2ω∗g11g20−2g112−g0223+g212,μ2=−Re⁡C10Re⁡λ′τ∗,β2=2Re⁡C10,T2=−Im⁡C10+μ2Im⁡λ′τ∗ω∗.

These quantities determine the properties of bifurcating periodic solutions. From the previous discussions, we have the following conclusions.

**Theorem 5 .**

Suppose that the conditions in (iii) of Theorem 4 hold, then the infected equilibrium *E*^*∗*^ undergoes a Hopf bifurcation at *τ* = *τ*^*∗*^, and *μ*_2_, *β*_2_, *T*_2_ determine the direction, stability, and period of the Hopf bifurcation, respectively,

- If *μ*_2_ > 0, a bifurcating periodic solution exists in the sufficiently small *τ*^*∗*^-neighbourhood.
- If *β*_2_ < 0 (*β*_2_ > 0), the bifurcating periodic solution is stable (unstable) when *t* → +*∞* (*t* → −*∞*).
- If *T*_2_ < 0 (*T*_2_ > 0), the period of the bifurcating periodic solution decreases (increases).

## 4. Simulations and Conclusions

For the main results in Sections 2 and 3, we now give some numerical simulations.

Based on the numerical simulations in [16, 27–29], take the following data:

(46) s=0.1,r=0.01,T=200,d=0.02,c=0.001,β=0.0027,δ=0.002,p=0.3,k=0.1,u=0.01.

**Figure 1:**
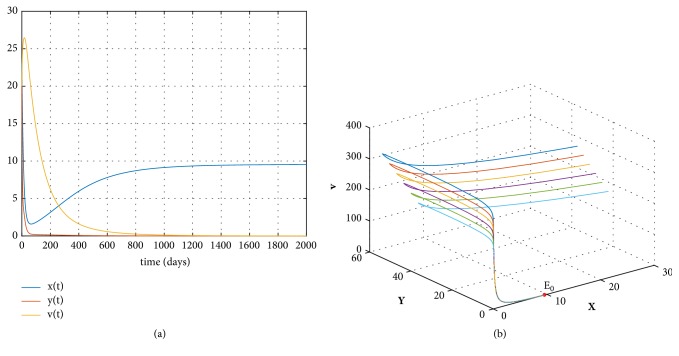
(a) The solution curves of the model (3) with *R*_0_ < 1. (b) The orbits of the model (3) when *R*_0_ < 1.

We can get *R*_0_ = 0.6363 < 1 and *E*_0_ = (9.5445,0, 0) by direct calculations. The uninfected equilibrium *E*_0_ is globally asymptotically stable by Theorem 2.  Figure 1 gives the curves and orbits of the model (3) with appropriate initial condition.

Furthermore, we also simulate the occurrence of Hopf bifurcations as the time delay *τ* increases. Take the following data:

(47) s=0.1,r=1.01,T=200,d=0.02,c=0.001,β=0.0027,δ=0.002,p=0.3,k=0.1,u=0.01.

**Figure 2:**
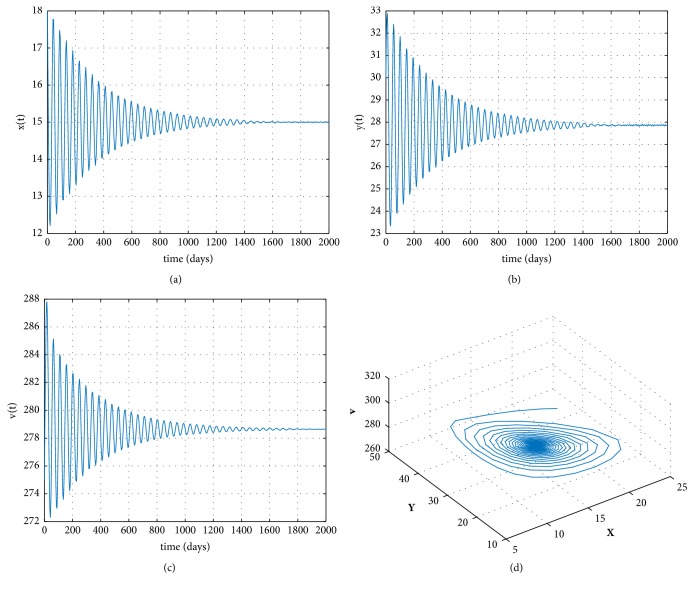
(a), (b), and (c) The solution curves of the model (3) with *R*_0_ > 1, *τ* = 10 < *τ*^*∗*^. (d) The orbits of the model (3) when *R*_0_ > 1, *τ* = 10 < *τ*^*∗*^.

**Figure 3:**
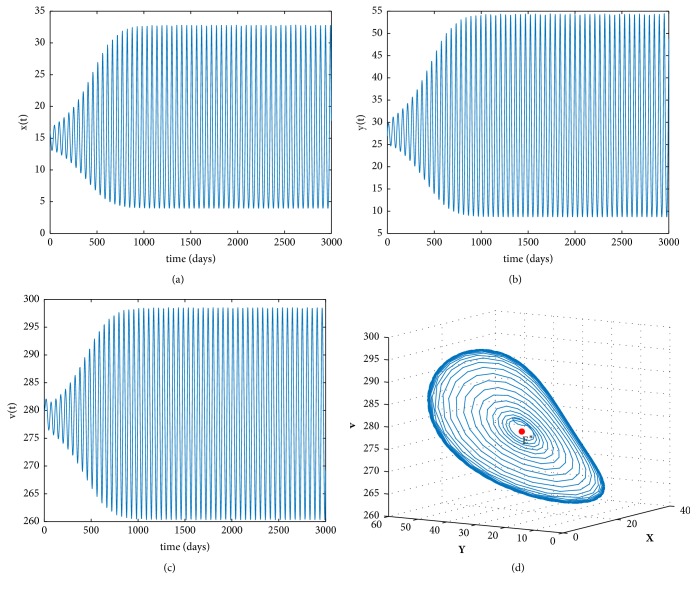
(a), (b), and (c) The solution curves of the model (3) with *R*_0_ > 1, *τ* = 12 > *τ*^*∗*^. (d) The orbits of the model (3) when *R*_0_ > 1, *τ* = 12 > *τ*^*∗*^.

By direct calculations, we get that (24) has a positive root *v*^*∗*^ = 0.0175 > 0, *R*_0_ = 13.0761 > 1, and *E*^*∗*^ = (15,27.8643,278.6435). And by simple computations, we attain *ω*^*∗*^ = 0.1322, *τ*^*∗*^ = 10.6528, and *h*′(*v*^*∗*^) = 0.0027 ≠ 0. From Theorem 4, the infected equilibrium *E*^*∗*^ is locally asymptotically stable when 0 < *τ* < *τ*^*∗*^ and unstable when *τ* > *τ*^*∗*^.  Figure 2 gives the stable phase trajectories and orbits of the model (3) when *τ* = 10 < *τ*^*∗*^.  Figure 3 gives the phase trajectories and orbits of model (3) when *τ* = 12 > *τ*^*∗*^ and it suggests that Hopf bifurcations occur. From (45), we obtain Re⁡(*C*_1_(0)) = −1.1035 × 10^−6^ < 0 for *τ* = 12. Therefore, both bifurcating periodic solutions are stable.

In this paper, we have proposed a delay HIV infection model (3) with a full logistic term *rx*(1 − (*x*(*t*) + *y*(*t*))/*T*). Then, using the basic reproduction number *R*_0_ = (*kδ*/*pu*)*x*_0_, we discuss the existence of the uninfected equilibrium *E*_0_ and the infected equilibrium *E*^*∗*^ = (*x*^*∗*^, *y*^*∗*^, *v*^*∗*^). By Routh-Hurwitz criterion, Liapunov-LaSalle invariance principle, and Hopf bifurcation method, we prove the following results.

If *R*_0_ ≤ 1, the uninfected equilibrium *E*_0_ is globally asymptotically stable when *τ* ≥ 0. That is to say, any solution (*x*(*t*), *y*(*t*), *v*(*t*)) trends to *E*_0_. Biologically, this means that the virus cannot successfully invade uninfected cells and will soon be cleared of the immune system. And as the time *t* increases, the virus will disappear. This suggests that we can control the disease by controlling the *R*_0_.

If *R*_0_ > 1, there exists a unique infected equilibrium *E*^*∗*^. The result of Theorem 4 implies that the time delay *τ* can destabilize the stability of the infected equilibrium *E*^*∗*^ and leads to the occurrence of Hopf bifurcations. And if *τ* ∈ [0, *τ*^*∗*^, the infected equilibrium *E*^*∗*^ is locally asymptotically stable. Biologically, this means that the HIV infection may become chronic. The infected equilibrium *E*^*∗*^ will be unstable and Hopf bifurcation occurs under some conditions when the time delay *τ* exceeds *τ*^*∗*^. In biology, this implies that the concentrations of uninfected cells, infected cells, and free virus will first tend to be constants and then oscillate as the time delay *τ* increases. In the immune response, this situation is very important for choosing the adequate drug treatment programs.

It can be found that the basic reproduction number *R*_0_ for the model (3) is not the same as that for model (2). It is independent on the constant *c*, which represents inhibitory effect on the growth of uninfected cells by infected cells. But *R*_0_ depends on the coefficient *r* of the full logistic term *rx*(1 − (*x*(*t*) + *y*(*t*))/*T*). Furthermore, the value of *x*^*∗*^ is independent on the coefficient *r*. And the values of *y*^*∗*^ and *v*^*∗*^ are the increasing functions with respect to *r*. And this paper shows that the time delay *τ* can produce richer dynamic behavior. As the time delay increases, the stability changes and periodic oscillations occur.

## Data Availability

The data used to support the findings of this study are available from the corresponding author upon request.
