# Corrigendum to “Modeling Inhibitory Effect on the Growth of Uninfected T Cells Caused by Infected T Cells: Stability and Hopf Bifurcation”

**DOI:** 10.1155/2019/7910208

**Published:** 2019-05-15

**Authors:** Yahui Ji, Wanbiao Ma, Keying Song

**Affiliations:** Department of Applied Mathematics, School of Mathematics and Physics, University of Science and Technology Beijing, 100083 Beijing, China

In the article titled “Modeling Inhibitory Effect on the Growth of Uninfected T Cells Caused by Infected T Cells: Stability and Hopf Bifurcation” [1], there were several errors throughout the article which are as follows:

- In the Abstract and the Introduction sections, “infection-free” should be corrected to “uninfected.”
- In the Local and Global Stability of the Equilibria section, there were the following errors:
  1. “We can denote the basic reproduction number of the HIV virus for the model (3) as *R*_0_=(*kδ*/*pu*)*x*_0_, $x_{0}=\left(T/2r\right)\left(r-d+\sqrt{{\left(r-d\right)}^{2}+4sr/T}\right)$ (see, for example, [3])” should be corrected to “We can denote the basic reproduction number of the HIV virus for the model (3) as *R*_0_=(*kδ*/*pu*)*x*_0_ (see, for example, [3]).”
  2. The sentence “Proof. We consider linear system of the model (3) in *E*_0_ near;” should be corrected to “Proof. We consider linear system of the model (3) at *E*_0_.”
  3. The sentence “When *R*_0_ < 1, *pu* − *kδx*_0_ ≠ 0. Therefore, *λ*=0 is not root of (9). If (8) has pure imaginary root *λ*=*iω*(*ω* > 0) for some *τ* > 0, substituting it into (8) and separating the real and imaginary parts,” should be corrected to “When *R*_0_ < 1, *pu* − *kδx*_0_ ≠ 0. Therefore, *λ*=0 is not root of (9). If (9) has pure imaginary root *λ*=*iω*(*ω* > 0) for some *τ* > 0, substituting it into (9) and separating the real and imaginary parts.”
  4. The sentence “Then, we get the corresponding *τ*_*k*_^(*n*)^ > 0 such that (23) has pure imaginary *λ*=*iω*_*k*_,” should be corrected to “Then, we get the corresponding *τ*_*k*_^(*n*)^ > 0 such that (19) has pure imaginary *λ*=*iω*_*k*_.”
  5. The sentence “From (19), we obtain *b*_2_*ω*^2^+*b*_3_^2^=(*ω*^3^ − *a*_2_*ω*)^2^+(*a*_1_*ω*^2^ − *a*_3_)^2^” should be corrected to “From (22), we obtain *b*_2_^2^*ω*^2^+*b*_3_^2^=(*ω*^3^ − *a*_2_*ω*)^2^+(*a*_1_*ω*^2^ − *a*_3_)^2^.”
  6. Equation (L) should be corrected as follows:

(L) dResλdτ−1λ=iωk=3vk3+2c1vk2+c2vkωk2b22ωk2+b32=vkh′vkωk2b22ωk2+b32.

(iii) In the Simulations and Conclusions section, the sentence “By direct calculations, we get that (19) has a positive root;” should be corrected to “By direct calculations, we get that (24) has a positive root.”
(iv) The formats of references 2, 14, 25, 26, 27, and 29 were updated.

This has been corrected in place.
